# Effects of Mg, Ca, Sr, and Ba Dopants on the Performance of La_2_O_3_ Catalysts for the Oxidative Coupling of Methane

**DOI:** 10.1021/acsomega.1c04738

**Published:** 2022-01-04

**Authors:** Danusorn Kiatsaengthong, Kanticha Jaroenpanon, Pooripong Somchuea, Thanaphat Chukeaw, Metta Chareonpanich, Kajornsak Faungnawakij, Hiesang Sohn, Günther Rupprechter, Anusorn Seubsai

**Affiliations:** † Department of Chemical Engineering, Faculty of Engineering, Kasetsart University, Bangkok 10900, Thailand; ‡ Center of Excellence on Petrochemical and Materials Technology, Kasetsart University, Bangkok 10900, Thailand; § Research Network of NANOTEC−KU on NanoCatalysts and NanoMaterials for Sustainable Energy and Environment, Kasetsart University, Bangkok 10900, Thailand; ∥ National Nanotechnology Center (NANOTEC), National Science and Technology Development Agency, Thailand Science Park, Khlong Luang, Pathum Thani 12120, Thailand; ⊥ Department of Chemical Engineering, Kwangwoon University, Seoul 01897, Korea; # Institute of Materials Chemistry, TU Wien, Vienna 1060, Austria

## Abstract

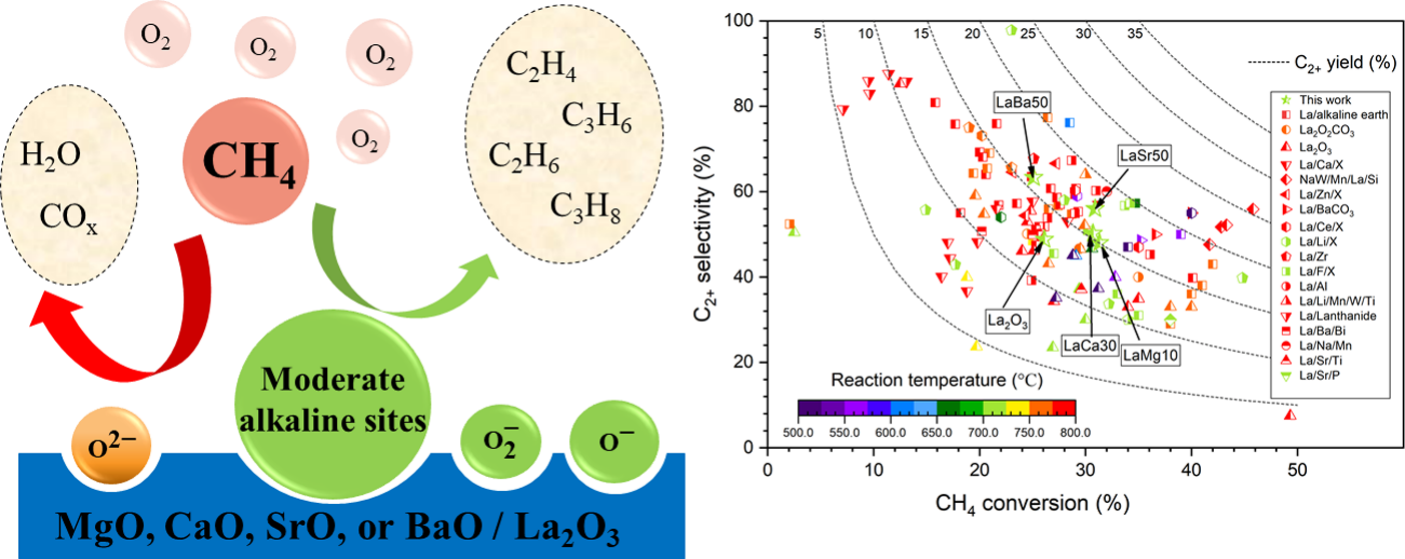

Oxidative coupling of methane (OCM) is a reaction to directly convert methane into high value-added hydrocarbons (C_2+_) such as ethylene and ethane using molecular oxygen and a catalyst. This work investigated lanthanum oxide catalysts for OCM, which were promoted with alkaline-earth metal oxides (Mg, Ca, Sr, and Ba) and prepared by the solution-mixing method. The synthesized catalysts were characterized using X-ray powder diffraction, CO_2_-programmed desorption, and X-ray photoelectron spectroscopy. The comparative performance of each promoter showed that promising lanthanum-loaded alkaline-earth metal oxide catalysts were La-Sr and La-Ba. In contrast, the combination of La with Ca or Mg did not lead to a clear improvement of C_2+_ yield. The most promising LaSr50 catalyst exhibited the highest C_2+_ yield of 17.2%, with a 56.0% C_2+_ selectivity and a 30.9% CH_4_ conversion. Catalyst characterization indicated that their activity was strongly associated with moderate basic sites and surface-adsorbed oxygen species of O_2_^–^. Moreover, the catalyst was stable over 25 h at a reactor temperature of 700 °C.

## 1 Introduction

Methane (CH_4_) is a chemical compound abundantly available in nature, which is mainly created from cultivation activities, livestock farming, mining combustion, and human/industrial waste.^1^ Methane can be used in industrial chemical processes and as fuel for ovens, homes, water heaters, kilns, automobiles, and turbines.^2^ Being the main component of natural gas, methane is important for electricity generation through combustion in gas turbines or steam generators. Moreover, it can be used to produce hydrogen gas via steam reforming of methane. In 2020, methane was used to produce over 52 million metric tons of hydrogen, which was used in petroleum refineries, chemical productions, and food industries.^3,4^ Moreover, there are several studies on using methane as a reactant to produce value-added chemicals via different methods.^5−7^ One of the prevalent reactions is the oxidative coupling of methane (OCM) over a suitable heterogeneous catalyst.

OCM, discovered by Keller and Bhasin in 1982,^8^ is a reaction to convert methane into high value-added hydrocarbons such as ethylene, ethane, propylene, and propane (denoted as C_2+_). However, byproducts such as carbon monoxide (CO) and carbon dioxide (CO_2_) are also produced. The proposed dominant mechanism involves dehydrogenation through methane activation by adsorbed oxygen species on the catalyst surface, forming methyl radicals. The coupling of two methyl radicals subsequently occurs to generate ethane molecules that can be dehydrogenated to ethylene. During the reaction, water may be formed as a byproduct. CO and CO_2_ can also originate from the nonselective oxidation of hydrocarbons.^9−11^

Several catalysts have been investigated in OCM, including rare earth oxide catalysts, which exhibited good activity due to the presence of reactive oxygen sites produced by surface oxygen vacancies. The reported catalysts provided C_2+_ yields >25% with 48–90% C_2+_ selectivities and 33–68% CH_4_ conversions at temperatures between 677 and 927 °C, which was still inefficient.^6,10,12−14^ This is due to the homogeneous gas-phase processes mainly controlling the coupling reaction. Therefore, at very high temperatures, the hydrocarbon yield is limited, regardless of the number of surface catalytic sites and methyl radicals. For this reason, highly efficient OCM catalysts should not only initiate the formation of free CH_3_ radicals at lower temperatures but also inhibit the nonselective oxidation of methane and hydrocarbons to CO_2_.^15^ Preventing CO*_x_* formation is, therefore, the major challenge of OCM.

Among OCM catalysts, lanthanum-based catalysts are of great interest that have also been modified with different promoters.^16−20^ In general, the addition of a catalyst promoter may improve specific properties, such as reducing the reaction temperature, increasing thermal stability, increasing basic sites or base strength, and modifying the mobility of lattice oxygen. Lanthanum oxides catalysts were first modified with alkaline by DeBoy and Hicks, who reported that 1 wt % alkaline-earth metal oxide could enhance the selectivity of lanthanum oxide catalysts: a La-Li catalyst had the highest C_2+_ selectivity of 75.9% with a 21.6% CH_4_ conversion.^21^

Song et al. reported that Sr-promoted La performed well at low temperatures (<500 °C) with excellent thermal stability.^19^ Jiang et al. reported that at lower temperatures, unpromoted lanthanide oxide nanorods outperformed lanthanum oxide nanopowders, nanosheets, and nanoflowers. Their best result was 14.8% C_2+_ yield with a 45.9% C_2+_ selectivity and a 32.3% CH_4_ conversion.^22^ Alkaline-earth metal oxide-promoted lanthanum oxide catalysts were also prepared without support. Uphade et al. and Choudhary et al. showed that unsupported lanthanum-promoted catalysts exhibited higher C_2+_ yields than those with supports.^23,24^

According to these reports, the basicity of the catalysts strongly influenced catalytic OCM activity. The addition of alkaline-earth metal oxides was reported to increase the basic sites and base strengths of catalysts, leading to an increase in C_2+_ formation. For example, Lim et al. used CaTiO_3_, SrTiO_3_, and BaTiO_3_ perovskite catalysts in OCM and found that SrO supported on BaTiO_3_ produced the highest C_2+_ yield (17.6% at a reaction temperature of 725 °C), resulting from strong surface basicity.^25^ Elkins et al. investigated different alkali and alkaline-earth metals (Li, Na, Mg, and Ca)-doped rare earth oxide (Sm_2_O_3_, TbO*_x_*, PrO*_x_*, and CeO_2_) catalysts supported on nanoparticle magnesium oxide (n-MgO) for OCM. They found that Li-TbO*_x_* supported on n-MgO outperformed all other prepared catalysts because of its stronger basic sites.^26^

One strategy to increase C_2+_ formation is to control the formation of CH_3_ radicals via creating surface electrophilic oxygen anions such as O^–^, O_2_^–^, O_2_^2–^, and O^2–^, as these species are crucial for C_2+_ or CO*_x_* formation. It has been reported that the surface O^2–^ lattice oxygen usually creates CO*_x_*, while O^–^, O_2_^–^, and O_2_^2–^ species generate C_2+_ products.^27^ Thus, some studies attempted to add alkaline-earth metal to lanthanum oxide catalysts because alkaline-earth metals usually act as oxygen-to-peroxide activators, which would create peroxide anions subsequently added to vacant sites in the lanthanum oxide lattice.

However, mechanistic details are still lacking and further studies are required, focusing on surface basicity, structural properties, and product optimization, which would be beneficial for future applications. Herein, (unsupported) lanthanum oxide mixed with oxides of alkaline-earth metal, including Mg, Ca, Sr, and Ba, are systematically investigated as OCM catalysts. Furthermore, the characterization of structure and adsorption properties enables us to understand the relationship between surface properties and catalytic performance.

## 2 Results and Discussion

### 2.1 Activity of Lanthanum Oxide Catalysts Loaded with Different Alkaline-Earth Metal Oxides

**Figure 1:**
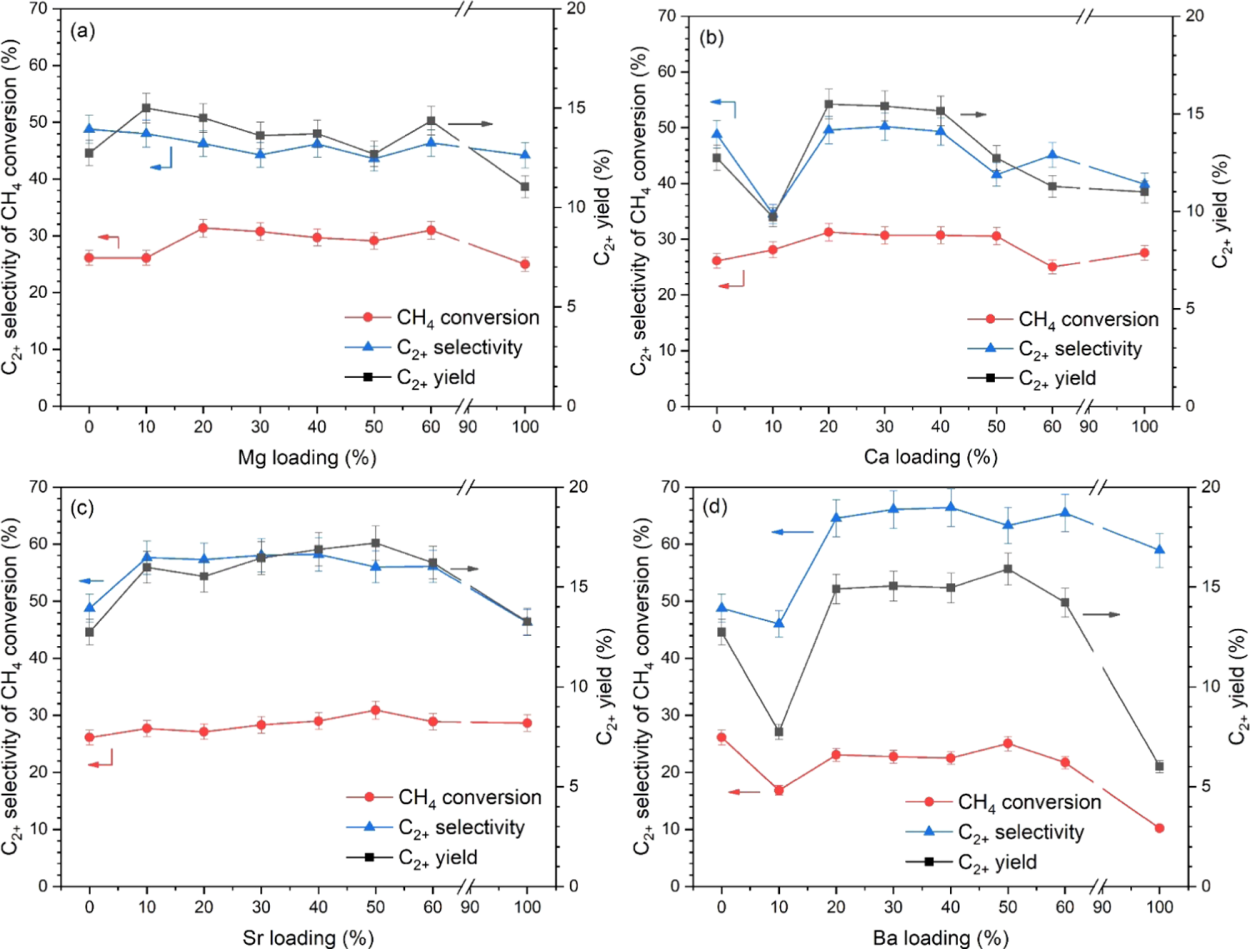
Effects of (a) Mg, (b) Ca, (c) Sr, and (d) Ba loading on La catalysts. Testing conditions: feed gas ratio of CH_4_/O_2_/N_2_ = 3:1:4, total feed flow rate = 35 mL min^–1^, total weight catalyst = 50 mg, and reactor temperature = 700 °C.

The kinetic performance of lanthanum oxide catalysts with different loadings (0–60 wt %) of alkaline-earth metal oxide (i.e., Mg, Ca, Sr, or Ba) was evaluated by the OCM reaction under identical conditions, as illustrated in Figure 1. The catalysts are defined as LaZY (where Z is the alkaline-earth metal (Mg, Ca, Sr, or Ba) and Y is its weight percentage on the catalyst). It was found that the lanthanum oxide catalyst exhibited a 12.7% C_2+_ yield with a 48.8% C_2+_ selectivity and a 26.1% CH_4_ conversion, while the pure oxides of Mg, Ca, Sr, or Ba had a lower C_2+_. The most promising alkaline-earth metal oxide-promoted lanthanum oxide catalysts were La-Sr and La-Ba, whereas La-Ca or La-Mg did not significantly improve the C_2+_ yield. The highest C_2+_ yield was observed for LaSr50, giving a C_2+_ yield of 17.2% with a 56.0% C_2+_ selectivity and a 30.9% CH_4_ conversion. The most active catalyst in the groups of Mg, Ca, and Ba was LaMg10 (15.0% C_2+_ yield), LaCa30 (15.4% C_2+_ yield), and LaBa50 (15.9% C_2+_ yield), respectively. Comparing the highest C_2+_ yields in each group, the catalysts were ranked as follows: LaSr50 > LaBa50 > LaCa30 > LaMg10.

**Figure 2:**
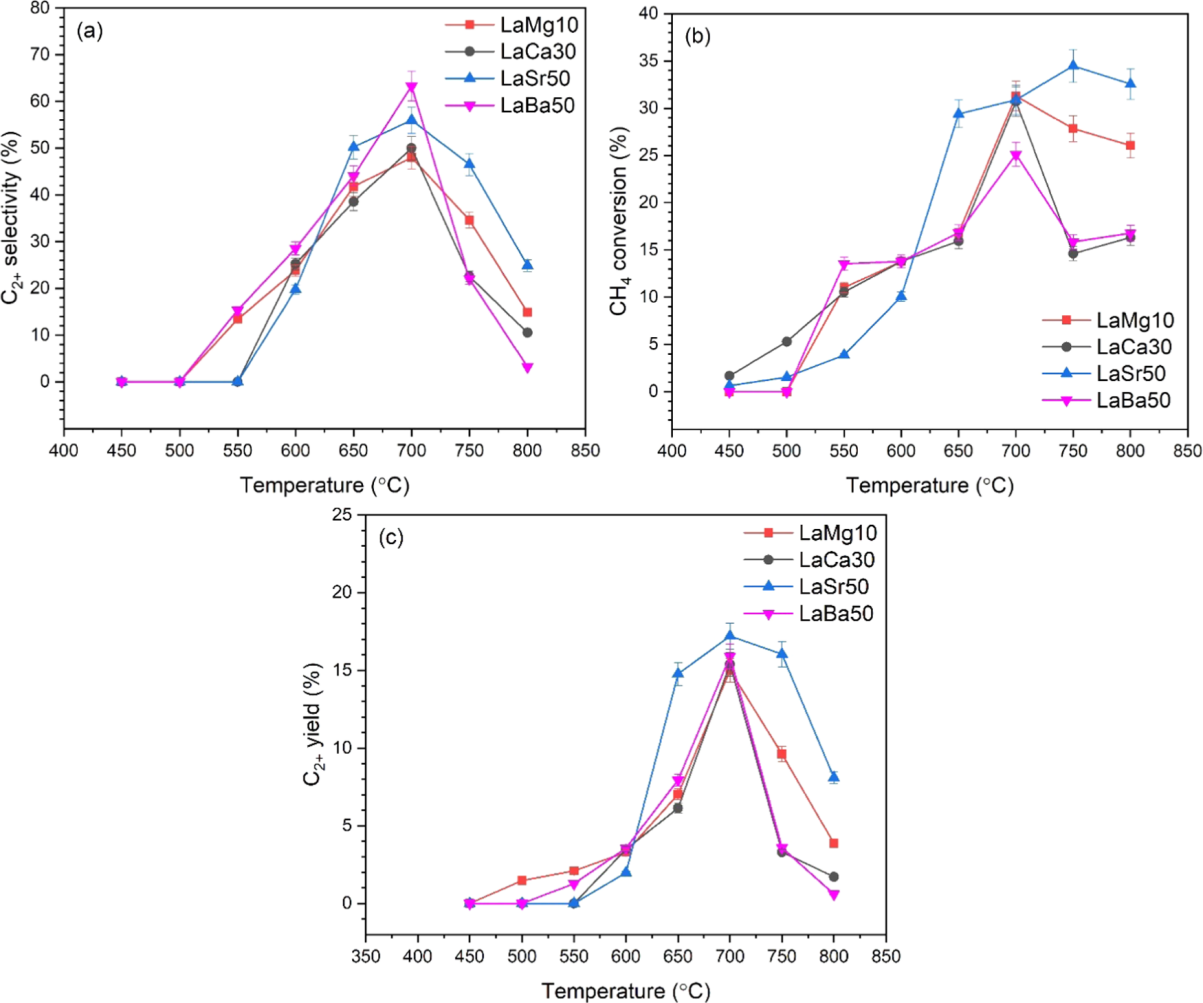
OCM performance of LaMg10, LaCa30, LaSr50, and LaBa50: (a) C_2+_ selectivity, (b) CH_4_ conversion, and (c) C_2+_ yield. Reaction conditions: feed gas ratio of CH_4_/O_2_/N_2_ = 3:1:4, total feed flow rate = 35 mL min^–1^, total weight catalyst = 50 mg, and reactor temperature = 450–800 °C.

The most active catalysts in the groups were further evaluated for OCM performance at different reactor temperatures (450–800 °C), as shown in Figure 2. All four catalysts were activated at approximately 500–550 °C; then, the performance of all four catalysts sharply increased to their maximum values at 700–750 °C. Interestingly, at 650 °C, LaSr50 exhibited much higher CH_4_ conversion and C_2+_ yield compared to the other three catalysts and kept exhibiting high performance until 750 °C, indicating that LaSr50 is superior to the other catalysts, especially in the reactor temperature range of 650–750 °C. At high reactor temperatures (750–800 °C), the C_2+_ selectivities of the catalysts dramatically decreased with a relatively small change of CH_4_ conversion, leading to a gradual decrease in the overall performance (i.e., C_2+_ yield). This could be because of the partial combustion of the products at high temperatures.^14^

### 2.2 Catalyst Characterization

#### 2.2.1 XRD Analysis

**Figure 3:**
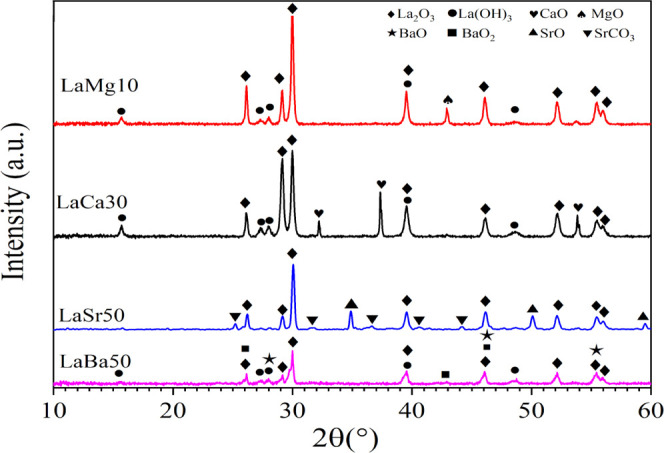
XRD patterns of fresh LaMg10, LaCa30, LaSr50, and LaBa50.

Based on the activity study in Section 2.1, the optimal lanthanum oxide catalysts from each group of alkaline-earth metal loading were LaSr50, LaBa50, LaCa30, and LaMg10. The crystal structures of the best lanthanum oxide catalysts were examined by X-ray diffraction (XRD), as depicted in Figure 3. Details on the diffraction peaks are collected in Table S1. XRD peak characteristics of La_2_O_3_ were observed for all catalysts. Moreover, XRD peaks of La(OH)_3_ were observed in LaMg10 and LaCa30 catalysts, which probably resulted from the interaction of La_2_O_3_ with moisture after calcination. XRD peaks indicating the presence of MgO, CaO, SrO, or BaO were observed for LaMg10, LaCa30, LaSr50, and LaBa50, respectively. SrCO_3_ and BaO_2_ were also detected but in small amounts. Nevertheless, the La_2_O_3_ phase interacting with the specific alkaline-earth metal oxide was considered as an active phase in the reaction.^17,28^ Interestingly, upon modification with alkaline-earth metal oxide, the crystallite size of La_2_O_3_ became smaller (see the crystallite size of La_2_O_3_ of each catalyst in Table S1), paralleled by an increase of C_2+_ yield. The crystallite sizes of La_2_O_3_ were: LaSr50 (21.9 nm) < LaBa50 (36.6 nm) < LaCa30 (37.0 nm) < LaMg10 (46.0 nm), inverse to the orders of C_2+_ yield. Note that the XRD patterns of the spent catalysts (after being used for 25 h) were also collected (Figure S1). It was observed that all of the XRD peaks of each spent catalyst did not considerably change compared to those of its fresh catalyst (Figure 3).

#### 2.2.2 N_2_ Sorption Analysis

The Brunauer–Emmett–Teller (BET) surface areas of both the pure fresh La and Sr catalysts and the fresh and spent bimetallic catalysts were evaluated using the nitrogen sorption–desorption technique (Table 1). The BET surface area of LaSr50 was 4.3 m^2^ g^–1^ compared to 2.3 m^2^ g^–1^ for the La catalyst and 0.4 m^2^ g^–1^ for the Sr catalyst. This correlates well with the XRD results (Figure 3) since smaller crystalline size occurs in the catalysts with larger surface areas. This could be because the second component (i.e., Sr) prevents the crystal growth of La_2_O_3_. LaMg10 showed a smaller BET surface area relative to the fresh one, which could be because of the aggregation of the catalyst particles. Note that the pore volume and pore size of all of the samples appeared to be undetectable because no hysteresis loops, i.e., nonporous materials, were found.

**Table 1:** BET Surface Area of the Fresh and Spent LaZY Catalysts

	BET surface area (m^2^ g^–1^)	
catalyst	fresh	spent
La	2.3	n/a
Sr	0.4	n/a
LaSr50	4.3	4.1
LaMg10	18.6	2.5
LaCa30	3.0	2.9
LaBa50	0.2	0.2

#### 2.2.3 CO_2_-TPD Analysis

**Figure 4:**
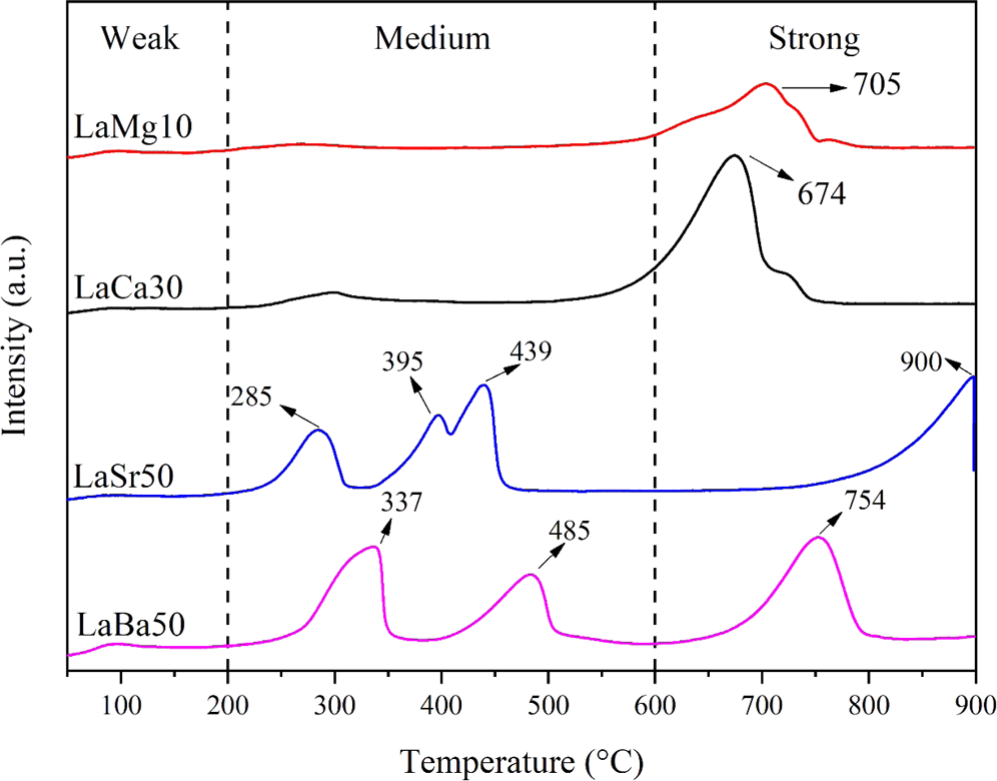
CO_2_-TPD profiles of LaMg10, LaCa30, LaSr50, and LaBa50.

The characterization of the optimal catalysts, including LaMg10, LaCa30, LaSr50, and LaBa50, was performed using CO_2_-TPD, as shown in Figure 4. This technique provides information on the nature of the surface basic sites of the catalysts. In CO_2_-TPD, CO_2_ desorption was measured in a temperature range of 50–900 °C. Generally, a peak that appears at the highest temperature indicates the highest base strength of the surface.^29^ In the CO_2_-TPD profiles, peaks were assigned to three temperature regions: below 200, 200–600, and above 600 °C, denoted as weak, moderate, and strong surface alkaline sites, respectively. It is widely known that moderate surface alkaline sites are crucial in improving C_2+_ selectivity.^10,16^ These active sites are generally inactive at low temperatures due to CO_2_ adsorption. As the reaction temperature increases, the CO_2_ molecules desorb and then the active sites are available to activate the CH_4_ molecules.^16^ For strong surface alkaline sites, they easily capture CO_2_ and convert it to stable carbonates that are inactive in the OCM reaction.^16,30^ Thus, it is reasonable to assume that the moderate surface alkaline sites play a crucial role in the OCM reaction. As illustrated in Figure 4 and by the relative peak areas in Table S2, the amount of the moderate surface alkaline sites of the catalysts followed the order: LaBa50 > LaSr50 > LaCa30 > LaMg10. As shown previously in Figure 1, the C_2+_ selectivity followed the order of the number of the moderate surface alkaline sites, indicating a correlation of the two.

#### 2.2.4 XPS Analysis

**Figure 5:**
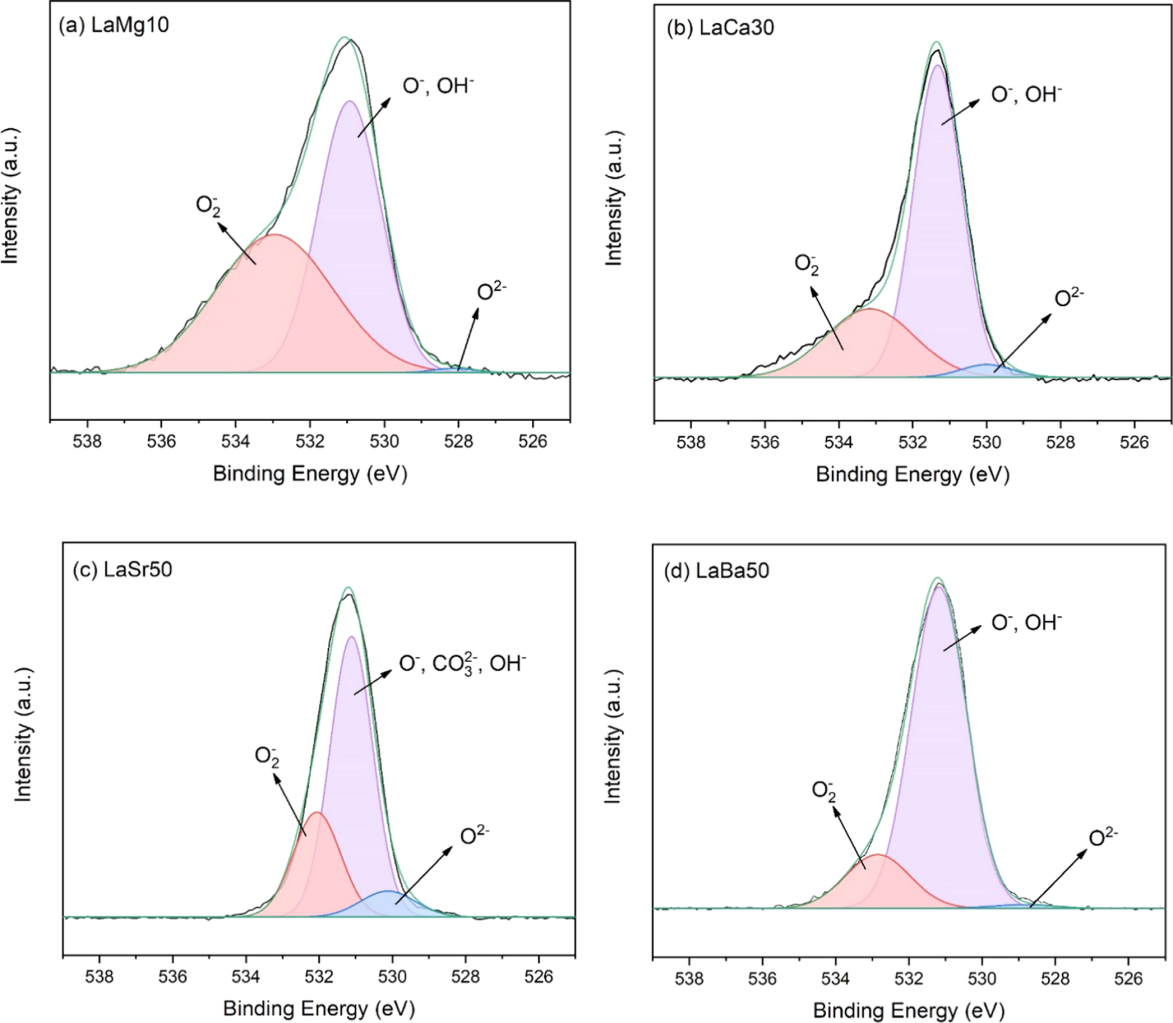
XPS O 1s spectra of (a) LaMg10, (b) LaCa30, (c) LaSr50, and (d) LaBa50.

The surface oxygen species of the optimal catalysts were further investigated using X-ray photoelectron spectrometry (XPS), as shown in Figure 5. These surface oxygen species in the O 1s region can be classified as follows: superoxides (O_2_^–^), peroxide ions (O^–^), hydroxide ions (OH^–^), carbonates (CO_3_^2–^), and lattice oxygens (O^2–^). Among these, the O_2_^–^ species are believed to play a crucial role in the activation of CH_4_.^16,17^ Thus, the relative amount of each specified oxygen species for each catalyst was determined to identify its correlation with the CH_4_ conversion, as summarized in Table 2. Note that (i) the CO_3_^2–^ species are only present in LaSr50 (Figure 5c) as was indicated by the XRD spectra (see Figure 3) and (ii) the binding energies of O^–^, OH^–^, and CO_3_^2–^ species are very close (approximately 531 eV).^17^ These species have been combined into one peak at 531 eV. The quantified XPS peaks of O_2_^–^ in Table 2 show that LaMg10 had the highest percentage fraction of O_2_^–^, followed by LaSr50, LaCa30, and LaBa50. These orders are consistent with the orders of CH_4_ conversion. Thus, the quantified peaks of O_2_^–^ species could be used to describe the performance of each catalyst in terms of activation of CH_4_.

**Table 2:** Curve-Fitted and Quantified XPS O 1s Peaks for LaMg10, LaCa30, LaSr50, and LaBa50 Catalysts

	O 1s binding energy (eV)/percentage fraction			
catalyst	O_2_^–^	O^–^, OH^–^, CO_3_^2–^	O^2–^	CH_4_ conversion (%)
LaMg10	533.5/48.2	530.8/51.1	528.1/0.7	31.3
LaCa30	533.4/27.4	531.3/65.0	530.2/7.6	30.7
LaSr50	532.2/28.9	531.1/62.8	530.1/8.3	30.9
LaBa50	533.0/40.4	531.2/53.8	528.9/5.8	25.1

### 2.3 Correlation of Catalyst Performance with Catalyst Properties

**Figure 6:**
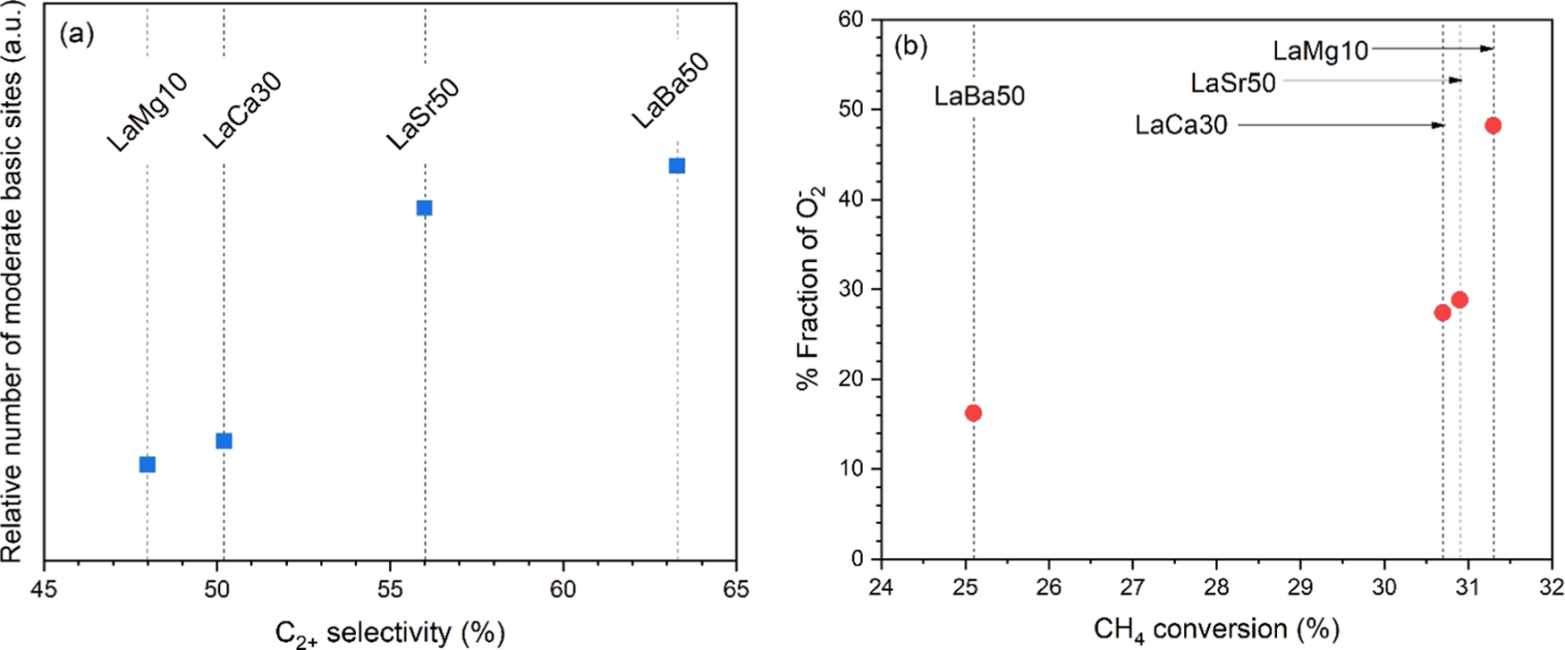
Plots of (a) the relative number of moderate basic sites vs C_2+_ selectivity and (b) % fraction of O_2_^–^ vs CH_4_ conversion.

The C_2+_ selectivity of each catalyst was plotted against the relative quantity of the moderate surface alkaline sites (Figure 6a), and the CH_4_ conversion of each catalyst was plotted against the percentage fraction of O_2_^–^ (Figure 6b). As observed in Figure 6a, the C_2+_ selectivity appears to improve when the quantity of the moderate surface alkaline sites increases, confirming that the moderate surface alkaline sites are essential in promoting the C_2+_ formation. In Figure 6b, the percentage fraction of O_2_^–^ also appears to correlate with the CH_4_ conversion, suggesting that CH_4_ conversion in the OCM reaction could be improved by designing a catalyst with a relatively high amount of O_2_^–^. Together, these plots suggest that a catalyst with a relatively high quantity of moderate surface alkaline sites and a relatively high amount of O_2_^–^ can be expected to exhibit a high C_2+_ yield.

### 2.4 Stability of the LaSr50 Catalyst

**Figure 7:**
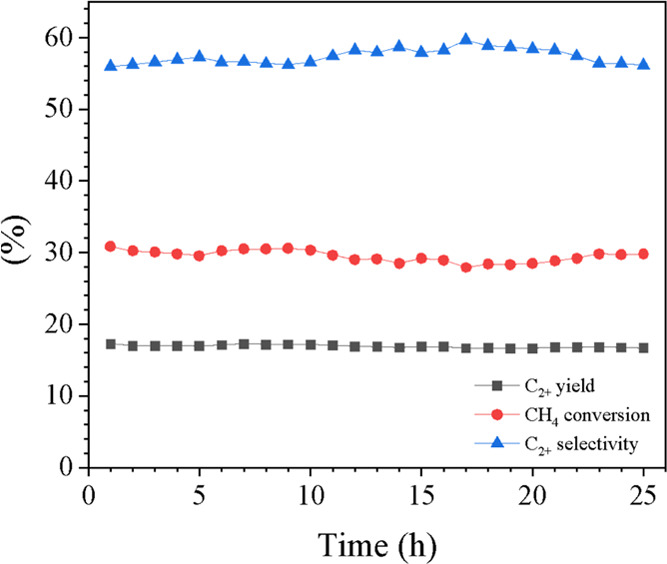
Time-on-stream result of LaSr50 catalyst over 25 h under a feed gas ratio of CH_4_/O_2_/N_2_ = 3:1:4, total feed flow rate = 35 mL min^–1^, total weight catalyst = 50 mg, and reactor temperature = 700 °C.

To determine the best catalyst, the stability of LaSr50 was tested over 25 h, as shown in Figure 7. The C_2+_ yield, C_2+_ selectivity, and CH_4_ conversion were maintained at approximately 17, 57, and 30%, respectively, throughout the entire period, with hardly any change in selectivity and conversion. This indicates that the catalyst is robust against deactivation.

### 2.5 Current La-Based Catalysts Compared to Others

**Figure 8:**
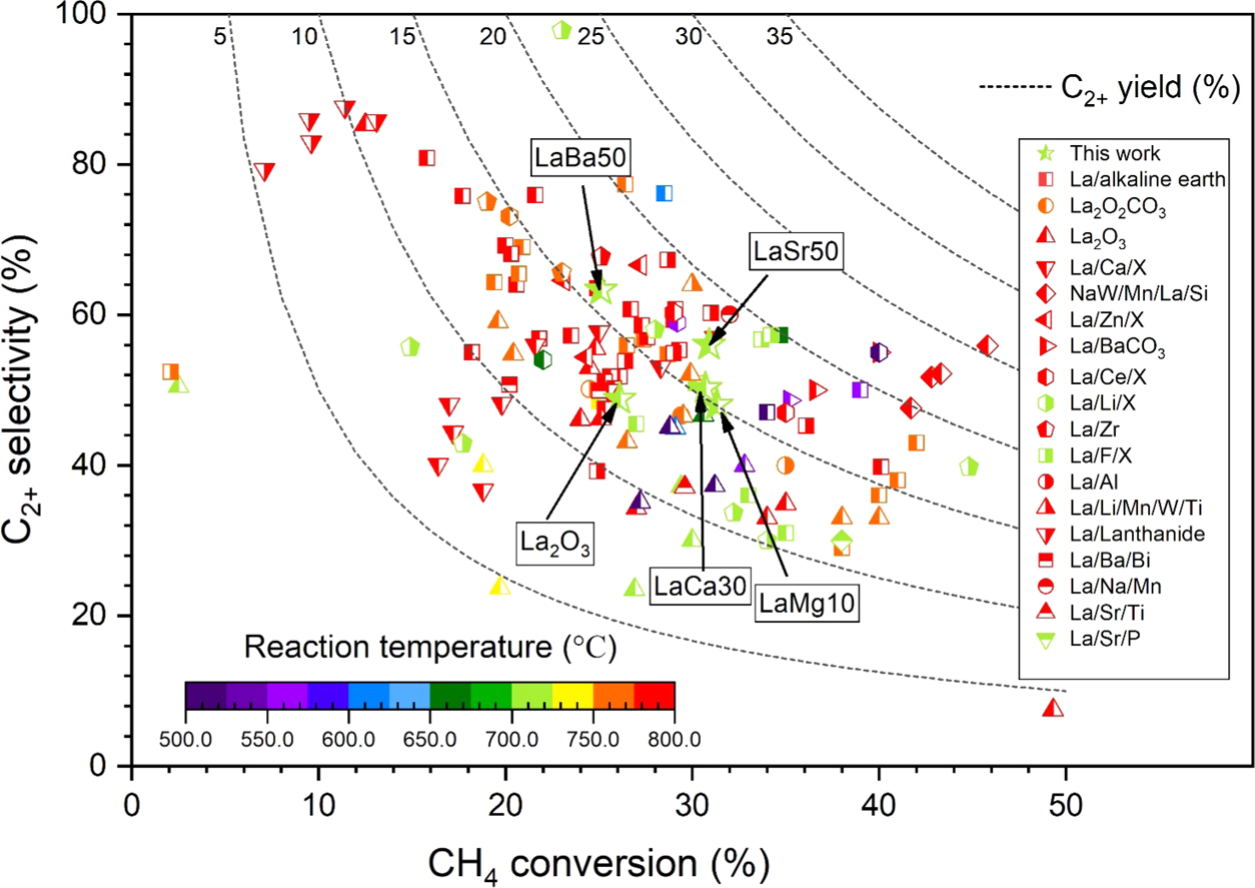
Survey of various La-based catalysts, including the current ones.

Figure 8 shows a review of various La-based catalysts for the OCM reaction that have been reported in the literature, and the details of each catalyst are described in Table S3. To be commercially viable, OCM catalysts should have a CH_4_ conversion of more than 30% and a C_2+_ selectivity of more than 80%.^31^ The catalysts investigated here fall outside of the commercial range. Several catalysts with a CH_4_ conversion of more than 30% have been observed, but their C_2+_ selectivity is below 80%. Most of the catalysts were operated in the reaction temperature range of 750–800 °C. Our current LaSr50 performed well at a lower temperature (700 °C), but more progress is required, particularly in increasing the C_2+_ selectivity to 80% while retaining CH_4_ conversion. Interestingly, a few reports showed that Li-La-Mg catalysts produced high C_2+_ selectivity (54–98%) but low CH_4_ conversion (22–24%) at a reaction temperature range of 650–700 °C.^32,33^ The goal of work in this area is to increase CH_4_ conversion rates, while maintaining this excellent C_2+_ selectivity and mild conditions. Accordingly, further investigation of the La-based catalysts is needed.

## 3 Conclusions

Alkaline-earth metal oxide-promoted lanthanum oxide catalysts were synthesized by the solution-mixing method. It is shown that alkaline-earth metal oxides, including MgO, CaO, SrO, and BaO, enhance the catalytic activity of the pure lanthanum oxide catalyst for OCM. The outstanding catalyst was LaSr50, followed by LaBa50, LaCa30, and LaMg10. The highest C_2+_ yield of 17.2% was obtained for LaSr50, with a 56.0% C_2+_ selectivity and a 30.9% CH_4_ conversion. The improvement of catalyst performance for C_2+_ formation is strongly associated with (i) a reduction of La_2_O_3_ crystallite sizes when each alkaline-earth metal oxide is added to the La_2_O_3_ catalyst, (ii) the presence of the moderate surface alkaline sites, and (iii) a high amount of O_2_^–^ species. Furthermore, the stability of LaSr50 was excellent, with no changes over 25 h. In situ studies are planned for the near future to gain further insight under rection conditions.

## 4 Material and Methods

### 4.1 Catalyst Preparation

Lanthanum(III) nitrate hexahydrate [La(NO_3_)_3_·6H_2_O, 99.00%, HiMedia], calcium nitrate [Ca(NO_3_)_2_·4H_2_O, 99.00%, Kemaus], magnesium nitrate hexahydrate [Mg(NO_3_)_2_·6H_2_O, 99.5%, QReC], strontium nitrate [Sr(NO_3_)_2_, 99.00%, HiMedia], and barium nitrate [Ba(NO_3_)_2_, 99.00%, HiMedia] were used as precursors for oxides of La, Ca, Mg, and Ba, respectively. The general procedure for preparing metal oxide catalysts is as follows. Each precursor was dissolved in ionized water to obtain the desired metal concentration. Weights of La and X (X = Mg, Ca, Sr, and Ba) were determined, and each metal solution was pipetted into a beaker to obtain a desired weight percentage of the metal components. The weight percentages of X ranged from 0 to 100 wt % on La. The atomic weight percentages of all of the prepared catalysts are shown in Tables S4–S7. After that, the mixture was stirred at room temperature for 1 h, followed by heating to 115 °C until dry. Then, the dried sample was calcined at 800 °C for 2 h in an air furnace with a heating rate of 5 °C min^–1^ and subsequently cooled down to room temperature.

### 4.2 Catalytic Activity Studies

The activity of each catalyst was evaluated in a plug flow reactor at atmospheric pressure, with the reactor temperature ranging from 450 to 800 °C. A sample (50 mg) was sandwiched between layers of quartz wool in a quartz tube reactor (an inner diameter of 0.5 cm with a length of 40 cm). The length of the catalyst bed was approximately 0.5 cm. The feed gas consisted of methane (CH_4_, 99.999%, Praxair), oxygen (O_2_, 99.999%, Praxair), and nitrogen (N_2_, 99.999%, Praxair) at a volume ratio of CH_4_/O_2_/N_2_ = 3:1:4.^13^ The inlet gas flow rate was 35 mL min^–1^, corresponding to a gas hourly space velocity (GHSV) of 13 400 h^–1^. All feed flow rates were controlled using mass flow controllers (Aalborg GFC17). The effluent was analyzed by gas chromatography using a flame ionization detector (FID; for quantifying C_2+_, including C_2_H_4_, C_2_H_6_, C_3_H_6_, C_3_H_8_, C_4_H_8_, and C_4_H_10_) and a thermal conductivity detector (TCD; for quantifying CO, CO_2_, and CH_4_). The activity of each catalyst was evaluated 1 h after the system reached the set point. Each experiment was performed a minimum of three times and plotted as an average value with error bars. Each standard gas was used to make a five-point calibration curve with a coefficient of determination (*R*^2^ > 0.99). A time-on-stream experiment over 25 h was conducted to monitor the catalysts’ stability. Equations 1–4 are used for calculating the % CH_4_ conversion, % C_2+_ selectivity, % CO*_x_* selectivity, and % C_2+_ yield, respectively.
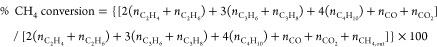
1
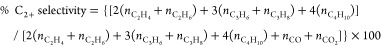
2
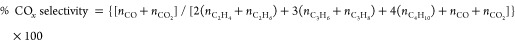
3
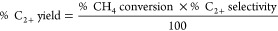
4

### 4.3 Catalyst Characterization

The crystal structure of the catalysts was analyzed using X-ray diffraction (PXRD; JEOL JDX-3530 and Philips X’Pert, using a step size of 0.02°, a step time of 0.5 s, and Cu Kα radiation at 45 kV and 40 mA). The average surface area of each catalyst was evaluated using an N_2_-adsorption analyzer (3Flex Physisorption Micromeritics), following the Brunauer–Emmett–Teller (BET) method at −196 °C. The basicity of the catalysts was analyzed using CO_2_-temperature-programmed desorption (CO_2_-TPD, Micromeritics’ AutoChem II 2920). In brief, each sample was pretreated under He flow at 120 °C for 1 h and cooled to 50 °C before 10% CO_2_/He mixed gas was flown over the catalysts for 1 h to adsorb onto basic sites. Any excess CO_2_ was flushed out by purging with He at 20 °C for 1 h. Then, the sample was heated to 900 °C at a heating rate of 10 °C min^–1^, while He was passed over the sample at 30 mL min^–1^. The amount of desorbed CO_2_ was monitored on stream using a TCD detector. The binding energy of each element in the catalysts was analyzed by X-ray photoelectron spectrometry (XPS, Kratos Axis Ultra DLD, using Al Kα radiation).

## Supporting Information Available

XRD patterns of spent catalysts; details of crystalline phases; relative number of CO_2_-TPD peaks; lanthanum-containing catalysts reported in the literature; and percentages by weight and atomic mass of catalysts (PDF)
