# Supplementary material for: Effects of Mg, Ca, Sr, and Ba Dopants on the Performance of La2O3 Catalysts for the Oxidative Coupling of Methane
Source: ACS Omega. 2022 Jan 4;7(2):1785–93. doi: 10.1021/acsomega.1c04738 (PMC8771708; doi:10.1021/acsomega.1c04738)
Supplement: Supplementary file 1 — ao1c04738_si_001.pdf [file ao1c04738_si_001.pdf]

Supporting Information for:

Effects of Mg, Ca, Sr, and Ba dopants on the performance of La_2O_3 catalysts for the oxidative coupling of methane

Danusorn Kiatsaengthong[1], Kanticha Jaroenpanon[1], Pooripong Somchuea[1], Thanaphat Chukeaw[1,2], Metta Chareonpanich[1,2,3], Kajornsak Faungnawakij[4], Günther Rupprechter[5], Hiesang Sohn[6], Anusorn Seubsai[1,2,3]*

[1] *Department of Chemical Engineering, Faculty of Engineering, Kasetsart University, Bangkok 10900, Thailand*

[2] *Center of Excellence on Petrochemical and Materials Technology, Kasetsart University, Bangkok 10900, Thailand*

[3] *Research Network of NANOTEC–KU on NanoCatalysts and NanoMaterials for Sustainable Energy and Environment, Kasetsart University, Bangkok 10900, Thailand*

[4] *National Nanotechnology Center (NANOTEC), National Science and Technology Development Agency, Thailand Science Park, Khlong Luang, Pathum Thani, 12120, Thailand*

[5] *Institute of Materials Chemistry, TU Wien, 1060 Vienna, Austria*

[6] *Department of Chemical Engineering, Kwangwoon University, Seoul 01897, Korea*

***Corresponding author**: fengasn@ku.ac.th

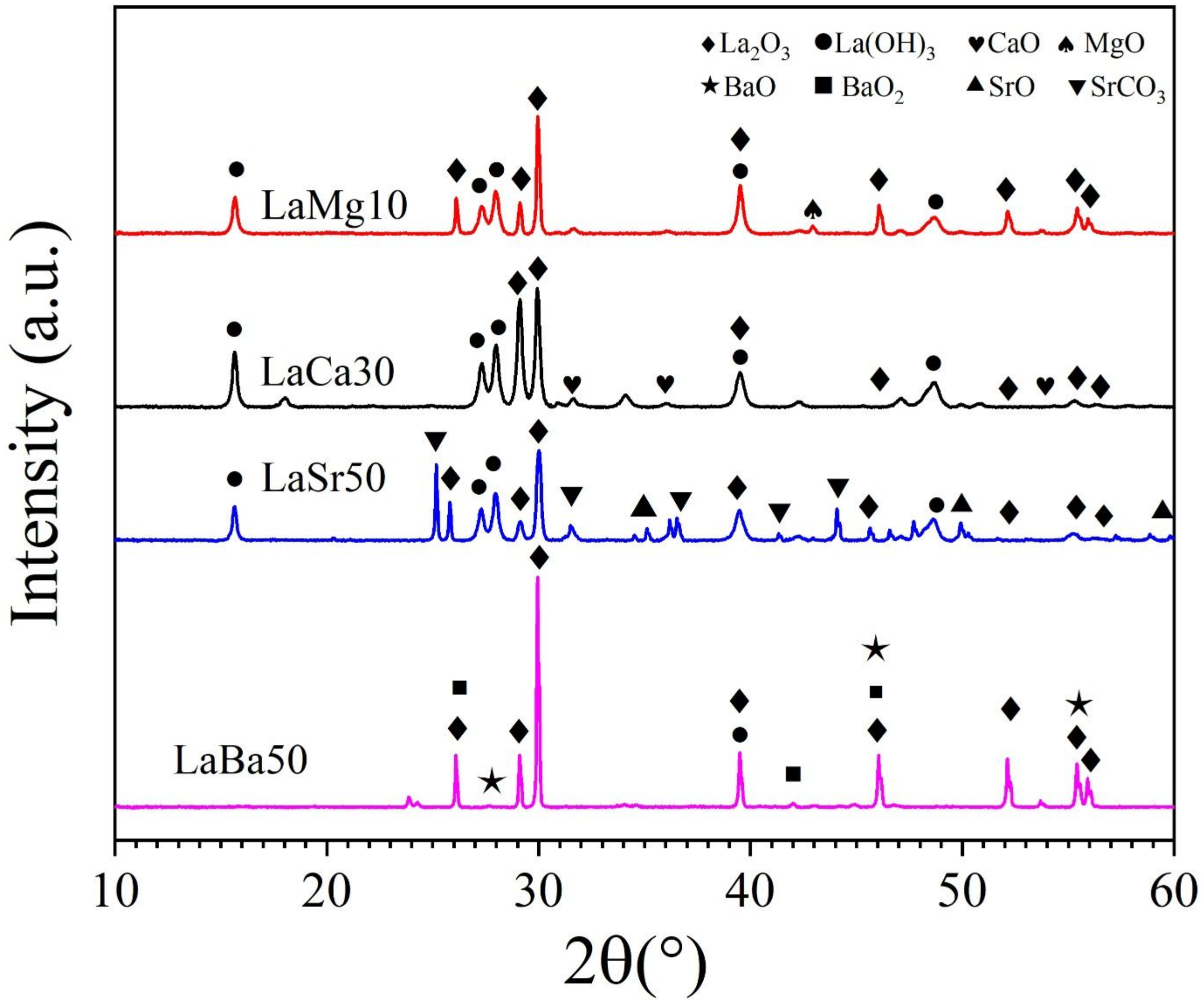

Figure S1. XRD patterns of spent LaMg10, LaCa30, LaSr50, and LaBa50.

Table S1. The crystalline phases of the best lanthanum oxide catalysts.

Catalyst	Crystalline structure	Symbol	Peaks	Crystallize size (nm)	Reference
10%LaMg	La_2O_3	♦	26.08, 29.08, 29.93, 39.46, 46.03, 52.08, 55.38, 55.88	46.0	(COD-1010278)
	$La(OH)_3$	●	15.86, 27.18, 28.03, 39.42, 48.57	28.3	(COD-4031381)
	MgO	♠	42.89		(COD-9000501)
30%LaCa	La_2O_3	♦	26.08, 29.08, 29.93, 39.46, 46.03, 52.08, 55.38, 55.88	37.0	(COD-1010278)
	$La(OH)_3$	●	15.86, 27.18, 28.03, 39.42, 48.57	30.7	(COD-4031381)
	CaO	♥	32.33, 37.48, 53.98		(COD-1011095)
50%LaSr	La_2O_3	♦	26.08, 29.08, 29.93, 39.46, 46.03, 52.08, 55.38, 55.88	21.9	(COD-1010278)
	SrO	▲	29.95, 34.77, 49.96, 59.35		(COD-7102430)
	$SrCO_3$	▼	25.20, 31.58, 36.55, 41.25, 44.07		(COD-9013802)
50%LaBa	La_2O_3	♦	26.08, 29.08, 29.93, 39.46, 46.03, 52.08, 55.38, 55.88	36.6	(COD-1010278)
	$La(OH)_3$	●	15.86, 27.18, 28.03, 39.42, 48.57	30.7	(COD-4031381)
	BaO	★	27.96, 46.19, 55.37		(COD-9008599)

Table S1. (Continue)

Catalyst	Crystalline structure	Symbol	Peaks	Crystallize size (nm)	Reference
50%LaBa	BaO_2	■	26.11, 42.65, 46.4		(COD-1521428)

Table S2. Relative number of CO_2- TPD peaks of LaZY catalysts.

Catalyst	Relative number of CO_2- TPD peaks (a.u.)			
	weak alkaline sites	moderate alkaline sites	strong alkaline sites	Total sites
LaMg10	0.8	2.1	6.2	9.0
LaCa30	0.6	2.6	9.8	13.0
LaSr50	0.7	7.6	6.3	14.6
LaBa50	1.2	8.5	7.9	17.6

Table S3. A summary of various Lanthanum-containing catalysts reported in literature.

Catalyst	Feature of the synthesized catalyst	Reaction temperature (ºC)	C_{2+} yield (%)	C_{2+} selectivity (%)	CH_4 conversion (%)	Reference
La_2O_3	Powder	700	12.7	48.8	26.1	This work
LaMg10	Powder	700	15.0	48.0	31.3	This work
LaCa30	Powder	700	15.4	50.2	30.7	This work
LaSr50	Powder	700	17.2	56.0	30.9	This work
LaBa50	Powder	700	15.9	63.3	25.1	This work
La_2O_3	Powder	800	13.0	53.0	24.5	[1]
Mg-La_2O_3	Powder	800	13.9	51.8	26.1	[1]
Ca-La_2O_3	Powder	800	13.3	51.8	25.6	[1]

Table S3. (Continue)

Catalyst	Feature of the synthesized catalyst	Reaction temperature (ºC)	C_{2+} yield (%)	C_{2+} selectivity (%)	CH_4 conversion (%)	Reference
Sr-La_2O_3	Powder	800	16.2	60.7	26.7	[1]
Ba-La_2O_3	Powder	800	13.2	57.2	23.5	[1]
La_2O_3	Powder	750	11.6	59.1	19.6	[2]
Li/ La_2O_3	Powder	800	16.4	75.9	21.6	[2]
Na/ La_2O_3	Powder	800	13.8	69.2	20.0	[2]
K/ La_2O_3	Powder	800	13.2	64.0	20.6	[2]
Mg/ La_2O_3	Powder	750	13.5	65.4	20.7	[2]
Ca/ La_2O_3	Powder	750	12.5	64.3	19.4	[2]

Table S3. (Continue)

Catalyst	Feature of the synthesized catalyst	Reaction temperature (ºC)	C_{2+} yield (%)	C_{2+} selectivity (%)	CH_4 conversion (%)	Reference
Sr/ La_2O_3	Powder	750	14.4	69.0	20.9	[2]
Ba/ La_2O_3	Powder	800	13.8	68.1	20.3	[2]
SrLaCe	Nanofiber	600	21.7	76.1	28.5	[3]
La-MgO	Powder	800	15.8	63.5	24.9	[4]
Sr-La_2O_3	Powder	800	17.2	59.3	29.0	[5]
Sr(5 wt %)/La_2O_3	Powder	800	10	55.0	18.2	[6]
$LiMgMnO_x/La_2O_3$	Powder	700	10.9	32.21	33.69	[7]
Sr-La_2O_3	Powder	800	16.2	60.7	26.7	[8]
Ca-La_2O_3	Powder	750	15.6	54.7	28.7	[8]

Table S3. (Continue)

Catalyst	Feature of the synthesized catalyst	Reaction temperature (ºC)	C_{2+} yield (%)	C_{2+} selectivity (%)	CH_4 conversion (%)	Reference
Ca-La_2O_3	Powder	726	12.1	48.5	25.0	[10]
$[La_5Cl_2(edta)_3 (H_2O)_{18}]_nCl_n$•$8nH_2O$	Powder	750	15.6	52.1	29.9	[11]
$[La_2(NO_3)_2(edta) (H_2O)_5]_n$•$3nH_2O$	Powder	650	14.3	46.7	30.6	[11]
$[La_2(SO_4)(edta) (H_2O)_3]_n$	Powder	750	11.4	43.2	26.5	[11]
$La_2O_2CO_3$	Powder	750	13.7	46.6	29.5	[12]
La_2O_3	Powder	750	13.7	41.9	32.7	[13]
Na-$LaMnO_3$	Powder	825	19.2	60.0	32.0	[14]
$Sr_{0.8}La_{0.2}TiO_3$	Powder	800	11.0	37.1	29.6	[15]

Table S3. (Continue)

Catalyst	Feature of the synthesized catalyst	Reaction temperature (ºC)	C_{2+} yield (%)	C_{2+} selectivity (%)	CH_4 conversion (%)	Reference
$BaCO_3/LaOF$	Powder	800	16.4	45.3	36.1	[16]
La_2O_3	Powder	850	13.8	55.5	24.8	[17]
La_2O_3	Powder	740	7.5	40	18.8	[18]
La_2O_3	Powder	760	13.2	33	40	[19]
La_2O_3	Powder	700	1.27	50.5	2.51	[20]
La_2O_3	Powder	775	11.6	46.2	25.1	[21]
Na/ La_2O_3	Powder	775	12.4	56.8	21.8	[21]
La_2O_3/MgO	Powder	875	16.2	39.8	40.1	[22]

Table S3. (Continue)

**Catalyst**	**Feature of the synthesized catalyst**	**Reaction temperature (°C)**	**C_{2+} yield (%)**	**C_{2+} selectivity (%)**	**CH_4 conversion (%)**	**Reference**
$BaF_2/LaOF$	Powder	780	19.3	67.3	28.7	[23]
La_2O_3	Powder	700	6.2	23.5	26.9	[24]
$LaLiO_2$	Powder	700	7.6	42.9	17.7	[24]
$(LaLiO_2)_{0.67}(CaO\text{-}MgO)_{0.33}$	Powder	700	17.8	39.8	44.8	[24]
$(LaLiO_2)_{0.67}(SrO\text{-}MgO)_{0.33}$	Powder	700	8.3	55.7	14.9	[24]
La_2O_3	Powder	725	4.7	23.7	19.7	[25]
Sr/ La_2O_3	Powder	880	12.8	80.8	15.8	[26]
La_2O_3	Powder	700	10.9	37.2	29.4	[27]

Table S3. (Continue)

**Catalyst**	**Feature of the synthesized catalyst**	**Reaction temperature (°C)**	**C_{2+} yield (%)**	**C_{2+} selectivity (%)**	**CH_4 conversion (%)**	**Reference**
SrF_2/ La_2O_3	Powder	700	19.6	57.3	34.2	[27]
SrO/ La_2O_3	Powder	700	12.3	45.5	27.0	[27]
BaO/ La_2O_3	Powder	800	12.0	47.5	25.3	[27]
BaF_2/ La_2O_3	Powder	800	14.2	53.8	26.4	[27]
LaOF	Powder	800	9.8	39.2	24.9	[27]
BaF_2/ LaOF	Powder	800	18.7	60.2	31.0	[27]
$LiMg_7La_{0.1}$	Powder	700	23.5	97.8	23.0	[28]
$Li_{4-6}LaTi$	Powder	750	14.2	75.0	19.0	[29]
$(LiLa)_{0.9}(MgSr)_{0.1}$	Powder	700	16.2	58.0	28.0	[30]

Table S3. (Continue)

Catalyst	Feature of the synthesized catalyst	Reaction temperature (°C)	C_{2+} yield (%)	C_{2+} selectivity (%)	CH_4 conversion (%)	Reference
$La_2Zr_2O_7$	Powder	750	15.1	65.6	23.0	[31]
La_2O_3-CaO	Powder	750	15.5	56.7	27.4	[32]
La/MgO	Powder	800	16.2	55.3	29.3	[33]
$(Sr_{0.2}La_{0.8})CeO$	Powder	750	14.8	73.1	20.2	[34]
La_2O_3	Powder	600	13.1	45.0	29.1	[35]
LaF_3	Powder	750	1.1	52.4	2.1	[35]
SrO/La_2O_3	Powder	700	15.4	50.9	30.2	[35]
SrO/LaF_3	Powder	700	19.1	56.7	33.7	[35]
SrF_2/La_2O_3	Powder	650	19.9	57.3	34.7	[35]

Table S3. (Continue)

**Catalyst**	**Feature of the synthesized catalyst**	**Reaction temperature (°C)**	**C_{2+} yield (%)**	**C_{2+} selectivity (%)**	**CH_4 conversion (%)**	**Reference**
La-CaO	Powder	800	15.7	57.0	27.6	[36]
La-CaO/SA-5552	Powder	800	8.2	48.1	17.0	[36]
La-CaO/SC-5532	Powder	800	6.6	40.1	16.4	[36]
La-CaO/SS-5231	Powder	800	9.5	48.2	19.8	[36]
La-CaO/SZ-5564	Powder	800	12.1	56.0	21.6	[36]
La-CaO/MgO/SA-5552	Powder	800	14.4	57.7	25.0	[36]
La-CaO/MgO/SC-5532	Powder	800	7.6	44.4	17.2	[36]
La-CaO/CaO/S A-5552	Powder	800	12.8	50.0	25.5	[36]

Table S3. (Continue)

**Catalyst**	**Feature of the synthesized catalyst**	**Reaction temperature (°C)**	**C_{2+} yield (%)**	**C_{2+} selectivity (%)**	**CH_4 conversion (%)**	**Reference**
La-CaO/CaO/S C-5532	Powder	800	6.9	36.7	18.8	[36]
La-CaO/La_2O_3/S A-5552	Powder	800	17.8	57.0	31.2	[36]
La-CaO/La_2O_3/S C-5532	Powder	800	15.0	53.1	28.3	[36]
La_2O_3	Powder	700	6.2	23.5	26.9	[37]
$LaLiO_2$	Powder	700	7.6	42.9	17.7	[37]
La_2O_3	Powder	775	12.2	34.9	35.0	[38]
$La(OH)_3$	Powder	775	11.2	33.0	34.0	[38]

Table S3. (Continue)

Catalyst	Feature of the synthesized catalyst	Reaction temperature (°C)	C_{2+} yield (%)	C_{2+} selectivity (%)	CH_4 conversion (%)	Reference
$LaAlO_3$	Powder	750	14.0	40.0	35.0	[38]
$La_2Ce_2O_7$	Powder	800	17.5	60.3	29.0	[39]
La_2O_3-Li-Mn/WO_3/TiO_2	Powder	750	19.2	64.0	30.0	[40]
La_2O_3	Powder	800	11.1	46.1	24.0	[41]
La_2O_3/MgO	Powder	800	12.9	51.1	25.3	[41]
$LaAlO_3$	Powder	750	12.3	50.1	24.5	[42]
Na_2WO_4/La–Mn/SiO_2	Powder	800	25.6	55.9	45.8	[43]
La/Na_2WO_4/Mn/SiO_2	Powder	800	19.8	47.6	41.7	[43]

Table S3. (Continue)

Catalyst	Feature of the synthesized catalyst	Reaction temperature (°C)	C_{2+} yield (%)	C_{2+} selectivity (%)	CH_4 conversion (%)	Reference
$Na_2WO_4/La/Mn/SiO_2$	Powder	800	22.1	51.7	42.8	[43]
$Na_2WO_4/Mn/La/SiO_2$	Powder	800	22.6	52.2	43.3	[43]
La_2O_3	Powder	850	10.7	85.3	12.5	[44]
La_2O_3–Nd_2O_3	Powder	850	11.2	85.8	13.1	[44]
La_2O_3–Sm_2O_3	Powder	850	10.0	87.6	11.4	[44]
La_2O_3–Gd_2O_3	Powder	850	8.2	85.9	9.5	[44]
La_2O_3–Er_2O_3	Powder	850	5.6	79.3	7.1	[44]
La_2O_3–Yb_2O_3	Powder	850	8.0	83.0	9.6	[44]

Table S3. (Continue)

**Catalyst**	**Feature of the synthesized catalyst**	**Reaction temperature (ºC)**	**C_{2+} yield (%)**	**C_{2+} selectivity (%)**	**CH_4 conversion (%)**	**Reference**
La_2O_3	Powder	750	12.0	33.0	38.0	[45]
Mg-La-O	Powder	750	11.0	29.0	38.0	[45]
Ca-La-O	Powder	750	14.0	36.0	40.0	[45]
Sr-La-O	Powder	750	15.0	38.0	41.0	[45]
Ba-La-O	Powder	750	18.0	43.0	42.0	[45]
La_2O_3	Powder	700	10.9	37.2	29.4	[46]
SrF2/La_2O_3	Powder	700	19.6	57.3	34.2	[46]
La_2O_3	Powder	800	3.7	7.5	49.3	[47]

Table S3. (Continue)

**Catalyst**	**Feature of the synthesized catalyst**	**Reaction temperature (°C)**	**C_{2+} yield (%)**	**C_{2+} selectivity (%)**	**CH_4 conversion (%)**	**Reference**
$La/BaCO_3$	Powder	800	18.3	50.0	36.6	[47]
La-MgO	Powder	800	13.4	75.8	17.7	[48]
La_2O_3	Powder	750	11.2	54.8	20.4	[49]
Sr/La_2O_3	Powder	750	12.9	56.0	23.2	[49]
Li/La_2O_3	Powder	780	17.0	67.7	25.1	[50]
La_2O_3	Powder	800	9.3	34.3	27.0	[51]
Ba_2LaBiO_6	Powder	800	12.5	49.9	25.0	[51]
$Ba_{0.7}La_{0.3}BiO_3$	Powder	800	10.2	50.7	20.2	[51]
La_2O_3/ZnO	Powder	800	13.2	54.4	24.3	[52]

Table S3. (Continue)

Catalyst	Feature of the synthesized catalyst	Reaction temperature (ºC)	C_{2+} yield (%)	C_{2+} selectivity (%)	CH_4 conversion (%)	Reference
$SrO-La_2O_3/ZnO$	Powder	800	14.9	64.6	23.1	[52]
$K_2O-La_2O_3/ZnO$	Powder	800	18.1	66.6	27.2	[52]
La_2O_3	Powder	550	13.1	40.0	32.8	[53]
$BaCO_3/La_2O_3$	Powder	550	17.1	48.6	35.2	[53]
La_2O_3	Powder	700	9.0	30.0	30.0	[54]
Li_3PO_4/La_2O_3	Powder	700	10.2	30.0	34.0	[54]
K_2SO_4/La_2O_3	Powder	700	10.8	31.0.	35.0	[54]
$SrHPO_4/La_2O_3$	Powder	700	11.4	30.0	38.0	[54]
$SrCl_2/La_2O_3$	Powder	700	11.9	36.0	33.0	[54]

Table S3. (Continue)

Catalyst	Feature of the synthesized catalyst	Reaction temperature (ºC)	C_{2+} yield (%)	C_{2+} selectivity (%)	CH_4 conversion (%)	Reference
$BaF_2/LaOF$	Powder	780	19.3	67.3	28.7	[55]
BaF_2/La_2O_3	Powder	780	15.9	54.9	29.0	[55]
BaO/La_2O_3	Powder	780	12.9	50.2	25.8	[55]
La_2O_3/CaO	Powder	750	14.8	55.9	26.5	[56]
La_2O_3/SrO	Powder	800	17.7	60.7	29.1	[56]
La_2O_3/BaO	Powder	800	16.0	58.6	27.3	[56]
Na/La_2O_3	Powder	750	20.4	77.3	26.4	[57]
$BaCO_3/LaOCl$	Powder	800	22.0	55.0	40.0	[58]
La_2O_3-CeO_2	Nanofiber	520	22.0	55.0	40.0	[59]

Table S3. (Continue)

**Catalyst**	**Feature of the synthesized catalyst**	**Reaction temperature (ºC)**	**C_{2+} yield (%)**	**C_{2+} selectivity (%)**	**CH_4 conversion (%)**	**Reference**
La_2O_3	Nanosheets	500	11.6	37.3	31.2	[60]
La_2O_3	Nanorod	500	13.0	45.1	28.8	[60]
La_2O_3	Nanoflower	500	9.5	35.1	27.2	[60]
Sr-La_2O_3	Nanofibers	500	16.5	47	35	[61]
$La_2Ce_2O_7$	Powder	800	17.2	59	29.2	[62]
La_2O_3-CeO_2	Nanofiber	570	18.0	70.0	25.7	[63]
Sr-La_2O_3	Microspheres	550	19.1	50.0	39.0	[64]
$La_2(Ce_{1-X}Mg_X)_2O_{7-\delta}$	Powder	650	11.9	54.0	22.0	[65]

Table S4. The percentages by weight and atomic mass of LaMg catalyst.

%wt		%mol	
**La**	**Mg**	**La**	**Mg**
100	0	100	0
90	10	61	39
80	20	41	59
70	30	29	71
60	40	21	79
50	50	15	85
40	60	10	90
0	100	0	100

Table S5. The percentages by weight and atomic mass of LaCa catalyst.

%wt		%mol	
**La**	**Ca**	**La**	**Ca**
100	0	100	0
90	10	72	28
80	20	54	46
70	30	40	60
60	40	30	70
50	50	22	78
40	60	16	84
0	100	0	100

Table S6. The percentages by weight and atomic mass of LaSr catalyst.

%wt		%mol	
**La**	**Sr**	**La**	**Sr**
100	0	100	0
90	10	85	15
80	20	72	28
70	30	60	40
60	40	49	51
50	50	39	61
40	60	30	70
0	100	0	100

Table S7. The percentages by weight and atomic mass of LaBa catalyst.

%wt		%mol	
**La**	**Ba**	**La**	**Ba**
100	0	100	0
90	10	90	10
80	20	80	20
70	30	70	30
60	40	60	40
50	50	50	50
40	60	40	60
0	100	0	100

References

[1] Vasant R. Choudhary; Shafeek A. R. Mulla; Rane, V. H. Surface basicity and acidity of alkaline eart promoted La_2O_3 catalysts and their performance in oxidative coupling of methane. *J. Chem. Technol. Biotechnol* **1998,** 72, 125-130.

[2] DeBoy, J. M.; Hicks, R. F. Oxidative coupling of methane over alkaline earth promoted La_2O_3. *J. Chem. Soc.* **1988,** 982-984.

[3] Sollier, B. M.; Bonne, M.; Khenoussi, N.; Michelin, L.; Miró, E. E.; Gómez, L. E.; Boix, A. V.; Lebeau, B. Synthesis and characterization of electrospun nanofibers of Sr-La-Ce oxides as catalysts for the oxidative coupling of methane. *Ind. Eng. Chem. Res.* **2020,** 59, 11419-11430.

[4] Vasant R. Choudhary; Shafeek A. R. Mulla; Uphade, B. S. Oxidative coupling of methane over supported La_2O_3 and La-promoted MgO catalysts: In fluence of catalyst-support interaction. *Ind. Eng. Chem. Res.* **1997,** 36, 2096-2100.

[5] Vasant R. Choudhary; Shafeek A. R. Mulla; Rane, V. H. Oxidative coupling of methane and oxidative dehydrogenaiton of ethane over strontium-promoted rare earth oxide catalysts. *J. Chem. Technol. Biotechnol* **1998,** 71, 167-172.

[6] Sollier, B. M.; Gómez, L. E.; Boix, A. V.; Miró, E. E. Oxidative coupling of methane on Sr/La_2O_3 catalysts: Improving the catalytic performance using cordierite monoliths and ceramic foams as structured substrates. *Appl Catal A Gen* **2017,** 532, 65-76.

[7] Li, Z.; He, L.; Wang, S.; Yi, W.; Zou, S.; Xiao, L.; Fan, J. Fast optimization of $LiMgMnO_x/La_2O_3$ catalysts for the oxidative coupling of methane. *ACS Comb. Sci.* **2017,** 19, 15-24.

[8] Vasant R. Choudhary; Balu S. Uphade; Mulla, S. A. R. Oxidative coupling of methane over a Sr-promoted La_2O_3 catalyst supported on a low surface area porous catalyst carrier. *Ing. Eng. Chem. Res* **1997,** 36, 3594-3601.

[9] Ana C. Ferreira, T. A. G., J.P. Leal, Joaquim B. Branco. Methane activation with nitrous oxide over bimetallic oxide Ca-lanthanide nanocatalysts. *Mol. Catal.* **2017,** 443, 155-164.

[10] Rane, V. H.; Chaudhari, S. T.; Choudhary, V. R. Comparison of the surface and catalytic properties of rare earth-promoted CaO catalysts in the oxidative coupling of methane. *J. Chem. Technol. Biotechnol.* **2006,** 81, 208-215.

[11] Yi-Chao Guo, Y.-H. H., Xin Dong, Yu-Chen Yang, Wen-Sheng Xia, Wei-Zheng Weng, Zhao-Hui Zhou. Well-defined lanthanum ethylenediaminetetraacetates as the precursors of catalysts for the oxidative coupling of methane. *Inorganica Chim. Acta* **2015,** 434, 221-229.

[12] Chen, M. H., Yuhui & Xia, Wen-Sheng & Weng, Wei-Zheng & Cao, Zexing & Zhou, Zhao-Hui & Wan, Hui-Lin. Dimeric 1,3-propanediaminetetraacetato lanthanides as the precursors of catalysts for the oxidative coupling of methane. *Dalton Trans.* **2014,** 43,

[13] Chen, M. G., Song & Zhou, Zhao-Hui. Isolations and characterization of highly water-soluble dimeric lanthanide citrate and malate with ethylenediaminetetraacetate. *Dalton Trans.* **2011,** 41,

[14] Shican Jiang, W. D., Kun Zhao, Zhen Huang, Guoqiang Wei, Yanyan Feng, Yijv Lv, Fang He. Enhanced chemical looping oxidative coupling of methane by Na-doped $LaMnO_3$ redox catalysts. *Fuel* **2021,** 299,

[15] Seoyeon Lim, J.-W. C., Dong Jin Suh, Kwang Ho Song, Hyung Chul Ham, Jeong-Myeong Ha. Combined experimental and density functional theory (DFT) studies on the catalyst design for the oxidative coupling of methane. *J. Catal.* **2019,** 375, 478-492.

[16] C.T. Au, Y. Q. Z., H. He, S.Y. Lai, C.F. Ng. The Characterization of $BaCO_3$-Modified LaOF Catalysts for the OCM Reaction. *J. Catal.* **1997,** 167, 354-363.

[17] V.R. Choudhary, V. H. R. Acidity/basicity of rare-earth oxides and their catalytic activity in oxidative coupling of methane to C_2-hydrocarbons. *J. Catal.* **1991,** 130, 411-422.

[18] S.J. Korf, J. A. R., J.M. Diphoorn, R.H.J. Veehof, J.G. van Ommen, J.R.H. Ross. The selective oxidation of methane to ethane and ethylene over doped and un-doped rare earth oxides. *Catal. Today* **1989,** 4, 279-292.

[19] Sławomir Kuś, M. O., Marian Taniewski. The catalytic performance in oxidative coupling of methane and the surface basicity of La_2O_3, Nd_2O_3, ZrO_2 and Nb_2O_5. *Fuel* **2003,** 82, 1331-1338.

[20] K. D. Campbell, H. Z., and J. H. Lunsford. Methane activation by the lanthanide oxides. *J. Phys. Chem.* **1988,** 92, 750-753.

[21] Youdong Tong, M. P. R., Jack H. Lunsford. The role of sodium carbonate and oxides supported on lanthanide oxides in the oxidative dimerization of methane. *J. Catal.* **1990,** 126, 291-298.

[22] Maria Traykova, N. D., Jeng-Shiang Tsaih, Alvin H. Weiss. Oxidative coupling of methane – the transition from reaction to transport control over La_2O_3/MgO catalyst. *Appl Catal A Gen* **1998,** 169, 237-247.

[23] Huilin Wan, Z. C., Weizheng Weng, Xiaoping Zhou, Junxiu Cai, Khirui Tsai. Constituent selection and performance characterization of catalysts for oxidative coupling of methane and oxidative dehydrogenation of ethane. *Catal. Today* **1996,** 30, 67-76.

[24] Masami Yamamura, H. O., Naohide Tsuzuki, Toshiya Wakatsuki, Kiyoshi Otsuka. Oxidative coupling of methane over ternary metal oxide catalysts consisting of Groups I, III and V elements in the periodic table. *Appl Catal A Gen* **1995,** 122, 135-149.

[25] Chiu Hsun Lin, K. D. C., Ji Xiang Wang, and Jack H. Lunsford. Oxidative dimerization of methane over lanthanum oxide. *J. Phys. Chem.* **1986,** 90, 534-537.

[26] Hubert Mimoun, A. R., Serge Bonnaudet, Charles J. Cameron. Oxypyrolysis of natural gas. *Appl Catal A Gen* **1990,** 58, 269-280.

[27] Wei Zheng Weng, R. L., Mingshu Chen, Xiaoping Zhou, Zisheng Chao, Hui Lin Wan. Study of the catalytic performance, surface properties and active oxygen species of the fluoride-containing rare earth-alkaline earth oxide based catalysts for the oxidative coupling of methane *Stud Surf Sci Catal* **1998,** 119, 343-348.

[28] Bi Yingli, Z. K., Jiang Yutao, Teng Chiwen, Yang Xiangguong. Catalytic oxidative coupling of methane over alkali, alkaline earth and rare earth metal oxides. *Appl Catal A Gen* **1988,** 39, 185-190.

[29] Gerald S. Lane, E. M., Eduardo E. Wolf. Methane oxidative coupling: II. A study of lithium-titania-catalyzed reactions of methane. *J. Catal.* **1989,** 119, 161-178.

[30] A. Kiennemann, R. K., A. Kaddouri, P. Poix, J.L. Rehspringer. Oxidative coupling of methane over $LnLiO_2$, $LnNaO_2$ and LnO_X catalysts (Ln = Sm, Nd, La ; X = Cl, Br). Promoting effect of MgO, CaO and SrO. *Catal. Today* **1990,** 6, 409-416.

[31] Xiuzhong Fang, L. X., Liang Peng, Yuan Luo, Junwei Xu, Luoji Xu, Xianglan Xu, Wenming Liu, Renyang Zheng, Xiang Wang. $Ln_2Zr_2O_7$ compounds (Ln = La, Pr, Sm, Y) with varied rare earth A sites for low temperature oxidative coupling of methane. *Chin. Chem. Lett.* **2019,** 30, 1141-1146.

[32] Vilas H. Rane, S. T. C., Vasant R. Choudhary. Oxidative coupling of methane over La-promoted CaO catalysts: Influence of precursors and catalyst preparation method. *J. Nat. Gas Chem.* **2010,** 19, 25-30.

[33] V.R Choudhary, V. H. R., S.T Chaudhari. Factors influencing activity/selectivity of La-promoted MgO catalyst prepared from La- and Mg-acetates for oxidative coupling of methane. *Fuel* 79, 1487-1491.

[34] Davi D. Petrolini, F. F. C. M., José M. Assaf, Elisabete M. Assaf. Statistical modeling applied to the oxidative coupling of methane reaction over porous (Sr_xLa_{1-x})CeO mixed oxides for optimization of C_2 yield, C_2 selectivity, and C_2H_4 selectivity. *CEJ. Advance* **2021,** 7,

[35] Rui-qiang Long, S.-q. Z., Ya-ping Huang, Wei-zheng Weng, Hui-lin Wan, Khi-rui Tsai. Promoting effect of F− on Sr/La oxide catalysts for the oxidative coupling of methane. *Appl Catal A Gen* **1995,** 133, 269-280.

[36] B.S. Uphade, S. A. R. M., V.R. Choudhary. Influence of metal oxide-support interactions in supported La-promoted CaO catalysts for oxidative coupling of methane. *Stud Surf Sci Catal* **1998,** 113, 1015-1021.

[37] A. Kaddouri, R. K., A. Kiennemann, P. Poix, J.L. Rehspringer. Oxidative coupling of methane over $LnLiO_2$ compounds (Ln = Sm,Nd,La). *Appl. Catal.* **1989,** 51, L1-L6.

[38] Yujin Sim, J. Y., Jeong-Myeong Ha, Ji Chul Jung. Oxidative coupling of methane over $LaAlO_3$ perovskite catalysts prepared by a co-precipitation method: Effect of co-precipitation pH value. *J. Energy Chem.* **2019,** 35, 1-8.

[39] Junwei Xu, L. P., Xiuzhong Fang, Ziyang Fu, Wenming Liu, Xianglan Xu, Honggen Peng, Renyang Zheng, Xiang Wang. Developing reactive catalysts for low temperature oxidative coupling of methane: On the factors deciding the reaction performance of $Ln_2Ce_2O_7$ with different rare earth A sites. *Appl Catal A Gen* **2018,** 552, 117-128.

[40] Fei Cheng, J. Y., Liang Yan, Jun Zhao, Huahua Zhao, Huanling Song, Ling Jun Chou. Enhancement of La_2O_3 to Li-Mn/WO_3/TiO_2 for oxidative coupling of methane. *J. Rare Earths* **2020,** 38, 167-174.

[41] Zhiming Gao, Y. S. Suppressed formation of CO_2 and H_2O in the oxidative coupling of methane over La_2O_3/MgO catalyst by surface modification. *J. Nat. Gas Chem.* **2010,** 19, 173-178.

[42] Gihoon Lee, I. K., Inchan Yang, Jeong-Myeong Ha, Hyon Bin Na, Ji Chul Jung. Effects of the preparation method on the crystallinity and catalytic activity of LaAlO3 perovskites for oxidative coupling of methane. *Appl. Surf. Sci.* **2018,** 429, 55-61.

[43] Jingjing Wu, H. Z., Song Qin, Changwei Hu. La-promoted Na_2WO_4/Mn/SiO_2 catalysts for the oxidative conversion of methane simultaneously to ethylene and carbon monoxide. *Appl Catal A Gen* **2007,** 323, 126-134.

[44] V.R Choudhary, S. A. R. M., B.S Uphade. Oxidative coupling of methane over alkaline earth oxides deposited on commercial support precoated with rare earth oxides. *Fuel* **1999,** 78, 427-437.

[45] Hiromi Yamashita, Y. M., Akira Tomita. Oxidative coupling of methane with peroxide ions over barium-lanthanum-oxygen mixed oxide. *Appl Catal A Gen* **1991,** 79, 203-214.

[46] Ruiqiang Long, Y. H., Weizheng Weng, Huilin Wan, Khirui Tsai. The performance and structure of rare earth oxides modified by strontium fluoride for methane oxidative coupling. *Catal. Today* **1996,** 30, 59-65.

[47] Zhao-Long Zhang, C. T. A., K.R. Tsai. Methane oxidative coupling to C_2 hydrocarbons over lanthanum promoted barium catalysts. *Appl. Catal.* **1990,** 62, L29-L33.

[48] V.R. Choudhary, V. H. R., S.T. Chaudhari. Surface properties of rare earth promoted MgO catalysts and their catalytic activity/selectivity in oxidative coupling of methane. *Appl Catal A Gen* **1997,** 158, 121-136.

[49] Z. Kalenik, E. E. W. Transient isotopic studies of the role of lattice oxygen during oxidative coupling of methane on Sr/La_2O_3 and Ca/ThO_2 Catalysts. *Stud Surf Sci Catal* **1993,** 75, 1093-1103.

[50] L. Wang, J. W., S. Yuan, Y. Wu. The active oxygen on the Li/La_2O_3 catalyst surface and its catalytic behavior in the oxidative coupling of methane. *Stud Surf Sci Catal* **1993,** 75, 2205-2208.

[51] A.K. Bhattacharya, S. C., K.K. Mallick, R.S. Talayan. Catalytic oxidative coupling of methane on metal oxides: I. Effect of oxidation state of bismuth and reversibility of lattice oxygen on activity in barium-lanthanum-bismuth oxides. *Appl Catal A Gen* **1992,** 85, 135-145.

[52] Xu Yide, Y. L., Guo Xiexian. Effect of basicity and adding CO_2 in the feed on the oxidative coupling of methane over K_2O and SrO promoted La_2O_3/ZnO catalysts. *Appl Catal A Gen* **1997,** 164, 47-57.

[53] Jiaxin Wang, L. C., Bing Zhang, Huanling Song, Jian Yang, Jun Zhao, Shuben Li. Low-temperature selective oxidation of methane to ethane and ethylene over $BaCO_3$/La_2O_3 catalysts prepared by urea combustion method. *Catal. Commun.* **2006,** 7, 59-63.

[54] J. Sanchez-Marcano, C. M., E.E. Wolf, G.A. Martin. Inhibition of the gas phase oxidation of ethylene by various solids and influence of their addition on the catalytic properties of lanthanum oxide towards the oxidative coupling of methane. *Catal. Today* **1992,** 13, 227-235.

[55] Zi Sheng Chao, X. P. Z., Hui Lin Wan, Khi Rui Tsai. Methane oxidative coupling on BaF_2LaOF catalyst. *Appl Catal A Gen* **1995,** 130, 127-133.

[56] X.-R. Xia, W. C., G.-X. Xiong, X.-X. Guo. Structure and performance of La_2O_3 promoted alkaline earth oxide catalysts for the oxidative coupling of methane. *Catal. Today* **1992,** 13, 617-620.

[57] J. Barrault, C. G., M. Hadj.Aissa, M. Dion, M. Tournoux. Oxidative coupling of methane over Na-La_2O_3 catalysts. *Catal. Today* **1990,** 6, 535-542.

[58] C.T. Au, H. H., S.Y. Lai, C.F. Ng. The oxidative coupling of methane overBa/CO_3LaOCl catalysts. *Appl Catal A Gen* **1997,** 159, 133-145.

[59] Noon, D.; Seubsai, A.; Senkan, S. Oxidative coupling of methane by nanofiber catalysts. *ChemCatChem* **2013,** 5, 146-149.

[60] Jiang, T.; Song, J.; Huo, M.; Yang, N.; Liu, J.; Zhang, J.; Sun, Y.; Zhu, Y. La_2O_3 catalysts with diverse spatial dimensionality for oxidative coupling of methane to produce ethylene and ethane. *RSC Adv.* **2016,** 6, 34872-34876.

[61] Song, J.; Sun, Y.; Ba, R.; Huang, S.; Zhao, Y.; Zhang, J.; Sun, Y.; Zhu, Y. Monodisperse Sr–La_2O_3 hybrid nanofibers for oxidative coupling of methane to synthesize C_2 hydrocarbons. *Nanoscale* **2015,** 7, 2260-2264.

[62] Xu, J.; Zhang, Y.; Xu, X.; Fang, X.; Xi, R.; Liu, Y.; Zheng, R.; Wang, X. Constructing $La_2B_2O_7$ (B = Ti, Zr, Ce) compounds with three typical crystalline phases for the oxidative coupling of methane: The effect of phase structures, superoxide anions, and alkalinity on the reactivity. *ACS Catal.* **2019,** 9, 4030-4045.

[63] Noon, D.; Zohour, B.; Senkan, S. Oxidative coupling of methane with La_2O_3–CeO_2 nanofiber fabrics: A reaction engineering study. *J. Nat. Gas Sci. Eng.* **2014,** 18, 406-411.

[64] Zhao, M.; Ke, S.; Wu, H.; Xia, W.; Wan, H. Flower-like Sr-La_2O_3 microspheres with hierarchically porous structures for oxidative coupling of methane. *Ind. Eng. Chem. Res.* **2019,** 58, 22847-22856.

[65] Petrolini, D.; Marcos, F.; Lucrédio, A.; Mastelaro, V.; Assafd, J.; Assafd, E. Exploiting oxidative coupling of methane performed over $La_2(Ce_{1-x}Mg_x)_2O_{7-\delta}$ catalysts with disordered defective cubic fluorite structure. *Catal. Sci. Technol*. **2021**, 11, 4471-4481.
